# Identifying early indicator traits for sow longevity using a linear-threshold model in Thai Large White and Landrace females

**DOI:** 10.5713/ajas.19.0855

**Published:** 2020-02-25

**Authors:** Suppasit Plaengkaeo, Monchai Duangjinda, Kenneth J. Stalder

**Affiliations:** 1 Department of Animal science, Faculty of Agriculture, Khon kaen University, Khon kaen, 40002, Thailand; 2 Department of Animal Science, 109 Kildee Hall, Iowa State University, Ames, IA 50011-3150, USA

**Keywords:** Genetic Parameter, Threshold Models, Litter Size, Sow Stayability, Swine

## Abstract

**Objective:**

The objective of the study was to investigate the possibility of utilizing an early litter size trait as an indirect selection trait for longevity and to estimate genetic parameters between sow stayability and litter size at different parities using a linear-threshold model for longevity in Thai Large White (LW) and Landrace (LR) populations.

**Methods:**

The data included litter size at first, second, and third parities (NBA1, NBA2, and NBA3) and sow stayability from first to fourth farrowings (STAY14). The data was obtained from 10,794 LR and 9,475 LW sows. Genetic parameters were estimated using the multiple-trait animal model. A linear-threshold model was used in which NBA1, NBA2, and NBA3 were continuous traits, while STAY14 was considered a binary trait.

**Results:**

Heritabilities for litter size were low and ranged from 0.01 to 0.06 for both LR and LW breeds. Similarly, heritabilities for stayability were low for both breeds. Genetic associations between litter size and stayability ranged from 0.43 to 0.65 for LR populations and 0.12 to 0.55 for LW populations. The genetic correlation between NBA1 and STAY14 was moderate and in a favorable direction for both LR and LW breeds (0.65 and 0.55, respectively).

**Conclusion:**

A linear-threshold model could be utilized to analyze litter size and sow stayability traits. Furthermore, selection for litter size at first parity, which was the genetic trait correlated with longevity, is possible when one attempts to improve lifetime productivity in Thai swine populations.

## INTRODUCTION

Sow longevity is a key productivity indicator for commercial pig breeding herds, involving sow productivity and the efficiency and profitability of the swine operation. Generally, the annual culling rate of sows is approximately 50% in commercial herds, of which 15% to 20% of culled sows can produce only one litter of piglets [1]. Koketsu [2] reported that sow culling at early parity had an influence on sow longevity. Stalder et al [3] suggested that a sow should produce at least three litters of piglets before being removed from the herd. Longevity traits are complex traits related to other traits such as body and leg structure [4] and number of pigs born alive (NBA) [5]. It would be beneficial to use these traits as predictive factors for lifetime performance and longevity. Furthermore, NBA has been reported as being an early indicator trait for lifetime performance and longevity [6], as high NBA in first parity tends to mean high NBA in subsequent parities [7]. However, the longevity trait is not expressed in early life; genetic associations between longevity and other economically important swine production traits are needed.

The genetic evaluation of animals for longevity traits presents a challenge in animal breeding. Because these traits are not normally distributed and the functional trait is recorded at the end of the female’s production life, they can be difficult to properly analyze when one is estimating genetic parameters and conducting genetic evaluations. In some countries, traditional methods of genetic evaluations based on a linear model are used for these traits; however, they have been reported as being unsuitable for nonlinear data [8]. Meanwhile, a nonlinear model, such as survival analysis, can provide a more accurate evaluation for these traits. Survival analysis has been widely used in the conducting of genetic evaluations for longevity traits in dairy cattle [9]. Multiple linear-threshold models (LTM) can handle litter size and longevity traits like stayability—which was defined as a binary trait—simultaneously [10]. Little published research has demonstrated the application of multiple-trait LTMs in the analysis of sow stayability and litter size across parities.

Ideally, the lifetime traits would be evaluated in the commercial female (Large White [LW]×Landrace [LR], F1). However, in most cases, the commercial female does not have a pedigree because pooled semen is used to generate replacement gilts. Without a pedigree, the genetic parameters cannot be estimated. In this situation, the best population in which to evaluate lifetime traits consists of purebred animals from the multiplication phase of production. It is important to obtain accurate estimates for the genetic association between litter size and longevity traits to improve the productivity of pigs using traditional and modern selection methods within the breeding scheme. Therefore, the objective of the study was to investigate the possibility of utilizing an early litter size trait as an indirect selection trait for longevity and to estimate genetic parameters between sow stayability and litter size at different parities using a LTM for longevity in Thai LW and LR populations.

## MATERIALS AND METHODS

### Animal care

No Institutional Animal Care and Use Committee (IACUC) approval was needed because the data used for this study was obtained from an existing database. However, details about gilt and sow management have been provided so that readers have this information with respect to typical Thai environmental conditions.

### Animal data, data editing, and trait definitions

Field data was accumulated from three farms in a large commercial production system located in the central and northeastern parts of Thailand. Data from the Sow Tracker Version 3.4.2 (Iowa Pork Industry Center, Iowa State University, Department of Animal Science, Ames, IA, USA) [11] reproductive data management software was extracted from each farm and combined into a new data set. The individual record for each sow included birthdate, age at first farrowing (AFF), first farrowing date, number of piglets born alive in each litter from the first to fourth litters, and sow culling date (including sows that were euthanized or that died of natural causes). Sow records that contained incomplete information or that were missing birth date, farrowing date, or parity, or for which the AFF was less than 280 days or greater than 460 days, were deleted from the dataset. After editing, the dataset contained 10,794 LR and 9,475 LW purebred sows born in the period 2004 to 2015. The summary of pedigree information from LR and LW sow populations is presented in Table 1. Contemporary groups were defined as the interaction of herd, year, and season of first farrowing. The months for a female’s first farrowing were categorized into three seasons: summer (March to June), rainy (July to October), and winter (November to February). Age at first farrowing was treated as a linear covariate. This study analyzed four traits. Litter size traits were defined as the number of piglets born alive in the first, second, and third parities (NBA1, NBA2, and NBA3, respectively). The stayability trait was defined as the stayability of the sow from the first to fourth parities (STAY14). The coding of the stayability trait was defined as the individual sow surviving up to the fourth parity (coded as 1) and a sow removed before the fourth parity (coded as 0). The reason for the use of the STAY14 trait in analysis is that a replacement gilt must produce at least three litters of piglets before the producer sees a positive cash flow [3].

### Management and nutrition

Replacement gilts were selected based on structural body conformation (feet, leg, underline, and external genitalia) [12] and the selection index that was based on the economic values and estimated breeding values for the reproductive traits (NBA) and growth traits (days to 104 kg and percent lean). Through all stages of the reproductive cycle, gilts and sows were housed in a facility that incorporated an evaporatively-cooled housing system (EVAP), with a partially slotted floor. Space allowance for each gilt was 2 m^2^, which met or exceeded National Pork Board [13] recommendations. The gilt’s body condition score (BCS) was evaluated using a five-point scoring system: emaciated (score 1), thin (score 2), ideal (score 3), fat (score 4), and obese (score 5) [14]. Gilts were fed according to their BCS, and the feed provided to individuals was increased or decreased for thin or excessively conditioned sows, respectively. Gilts were inseminated at approximately 35 weeks of age. Estrus was detected twice daily at 8.00 and 16.00 by experienced staff and using a vasectomized boar. The vasectomized boar was used to stimulate observable signs of estrus and to facilitate heat detection. Gilts and sows were mated using artificial insemination, totaling two or three times per estrus. After mating, gilts and sows were housed in individual stalls in EVAP throughout their entire gestation periods. Approximately five to seven days before farrowing, gilts and sows were moved from gestation units to farrowing units. The farrowing stalls (2.2 m× 3.6 m) had a partially slotted floor, a creep area that provided supplementary heat through a heated lamp (partitioned corner of the pen) for the piglets, and an anti-crush bar to reduce pre-weaning mortality (crushing of piglets by the sow). Replacement gilts and sows were fed 1.8 to 2.0, 2.0 to 2.4, and 3 kg feed/d (14% crude proteins and 2,950 kcal/kg metabolizable energy) during weeks three through twelve of gestation (adjusted for BSC as previously described) and until three days before expected farrowing, respectively. After farrowing, lactating sows were fed *ad libitum* (full feeding) from the day of farrowing on. All gestation and farrowing diets met or exceeded National Research Council [15] recommendations for each phase of production. Pigs were weaned at 19 to 21 days of age.

### Statistical analysis

Genetic parameters were estimated by the multiple-trait animal model. A LTM was used in which NBA1, NBA2, and NBA3 were continuous traits and STAY14 was considered a binary trait. The following linear-threshold animal model was used in the analysis:

y=Xβ+Za+e,

where *y* is the vector of observations of the actual observed value for NBA1, NBA2, and NBA3, and the unobservable liability for STAY14; *β* is the vector of fixed effects (AFF was treated as covariates, farm-year-season at first farrowing for litter traits and stayability traits); *a* is the vector containing random additive genetic effects for the animal; *e* is the vector of random residual effects; and *X* and *Z* are the correspondent incidence matrix of the fixed effects and additive genetic, respectively. The distribution of *a* and *e* was assumed to be normal, with zero means and variances following *a*~*N*(0,*A* ⊗ *G*) and *e*~*N*(0,*I* ⊗ *R*), respectively, where *A* is the numerator relationship matrix, *G* and *R* are the additive genetic and residual variance-covariance matrix across four traits, *I* is an identity matrix, and ⊗ is the Kronecker product operator. For binary data, the parameters were not identifiable. Therefore, the residual variance was fixed to 1 and the threshold was set to 0, which is a standard assumption in categorical data analysis [16].

The (co)variance components and genetic parameters were estimated utilizing Bayesian inference methods that were implemented using the program THRGIBBS1F90 [17], with a linear-threshold animal mixed model allowing for any combination of continuous and categorical traits. The analysis was run as a single chain of 250,000 cycles with a burn-in period for the first 50,000 cycles; every 50th sample was stored thereafter, for a total of 4,000 samples used for parameter estimation. Posterior means and standard deviation estimates were obtained using POSTGIBBSF90 [17].

## RESULTS AND DISCUSSION

The data description for the four traits evaluated in the present study is presented in Table 2. Average AFF was 367±26.0 days and 377±27.3 days for LR and LW sows, respectively. In the present study, litter size at birth increased from parities 1 to 3 for both LR and LW sow populations, which is in agreement with previous findings [18,19]. However, LW sows had a larger litter size at birth as compared to LR sows at parities 1 to 3.

### Heritabilities

Heritability and variance component estimates based on posterior means are shown in Table 3. Heritability estimates were low for litter traits from both LW and LR populations, which is in accordance with previous literature reports [18,20]. The heritability estimates for litter size at birth tended to increase as parity increased from 1 to 3 (0.02±0.01 to 0.06±0.01) for LW sows. In contrast, the heritability estimates for litter size at birth from LR sows slightly increased from Parity 1 to Parity 2, and then were constant from Parity 2 to Parity 3 (Table 3). Roehe and Kennedy [21] reported similar increasing trends for litter size heritability estimates (0.08 to 0.14) as parity increased from 1 to 4 in Canadian Yorkshire and LR sows. This result indicates that the multivariate model could be adapted by treating the number of piglets born alive at different parities as different traits [22].

The heritability estimates for the stayability trait in the present study were low: 0.05±0.01 in LR sows and 0.05±0.01 in LW sows. Additionally, that been reported the heritabilities for stayability tend to increase with parity up to Parity 3 [22]. In the present findings, the STAY14 heritability estimates were lower in both breeds (Table 3). These estimates are in agreement with previous literature estimates. López-Serrano et al [22] reported low heritability estimates for the stayability trait (first to third litters) in LW and LR sows. Similarly, Engblom et al [18] reported low heritability estimates from the threshold model for stayability from crossbred LR×LW sows. Moreover, Engblom et al [18] reported that larger heritability estimates for stayability traits were obtained when estimated using a threshold model as compared to estimates obtained when a linear model was implemented. Such heritability estimate differences may be a result of population structure, genetic background, and environmental and management conditions.

### Genetic and phenotypic correlations

Posterior mean estimates for genetic and phenotypic correlations (±standard error) between litter size at different parities are presented in Table 4 and 5 for LR and LW sows, respectively. All genetic correlations between litter size across parities ranged from 0.73±0.13 to 0.89±0.05 in LR sows and from 0.72±0.01 to 0.92±0.02 in LW sows. Relatively large genetic relationships between NBA1, NBA2, and NBA3 were observed in the present study. Data from the present study indicated that litter size at different parities seems to be genetically associated but with heterogeneous variances (Table 3). The findings from the present study agree with other estimates reported in the scientific literature [20,23] and indicate that litter size at birth in the first parity should be regarded as a different trait from litter size at birth from subsequent parities. Therefore, when one is conducting a genetic evaluation of those traits, a multiple-trait model should be considered an effective alternative method. Phenotypic correlations between litter size across parities were low (Tables 4, 5).

The genetic and phenotypic correlation estimates between litter size and the stayability trait are shown in Table 4 and 5 for LR and LW sows, respectively. Genetic correlation estimates between litter size and the stayability trait for LR ranged from 0.43±0.18 to 0.65±0.16, while for LW they ranged from 0.12±0.01 to 0.55±0.01. On the other hand, a moderate and favorable relationship between NBA1 and STAY14 for both LR and LW sows was observed. In the present study, positive genetic correlations between NBA1 and STAY14 were observed for LR and LW sows. Although the genetic correlation between NBA2 and STAY 14 tended to differ, the direction of the genetic correlations was the same. Since the genetic correlation involves the (co)variation of the trait in the population, estimates for genetic correlation between 2 traits may differ between populations for a variety of reasons including population size, the length of time that selection has been practiced for the traits, different genetic background, etc. This result was consistent with that of the previous study of Engblom et al [24], which reported a small but positive genetic relationship among NBA1 and STAY12 and STAY13, ranging from 0.03 to 0.07 in Swedish crossbred sows. This favorable relationship suggests that sows with high breeding values for litter size at first parity tend to have a high genetic potential to stay in the herd until Parity 4. Therefore, litter size at first parity is a potential early indicator trait for longevity. The selection emphasis on litter size at Parity 1 may have a desirable impact on sow stayability in the present study.

However, Tholen et al [25] reported a negative genetic relationship between litter size in the first litter and the stayability of the sow from the first to the fourth parities, ranging from −0.04 to −0.25 in a synthetic line. Commercial farms in Thailand use semen from different boars in the two or three inseminations per heat period, which causes a lack of pedigrees for commercial F1 females. In addition, purebred animals have phenotypic records for lifetime measurements and animals can express their lifetime potential. Therefore, the best population in which to evaluate longevity traits is the purebred animals from the multiplication phase of production.

In conclusion, a LTM could be utilized to analyze litter size and sow stayability traits. Selection for litter size at first parity as a potential early indicator trait, which was the genetic trait correlated with longevity, is possible when one is attempting to improve lifetime productivity. Therefore, efforts to improve sow productivity through a genetic evaluation program in a commercial pork system should include both litter size and sow longevity.

## Figures and Tables

**Table 1:** Summary of pedigree information from Thai Landrace and Large White sow populations

Item	Landrace	Large White
Number of base animals	12,760	11,223
Number of animals with record	10,749	9,475
Number of animals with unknown sire	1	0
Number of animals with unknown dam	1	0
Number of animals with both parents unknown	0	1
Number of sires with progeny records	412	433
Number of dams with progeny records	3,068	2,975

**Table 2:** Summary statistics for litter size and stayability traits from Thai Landrace and Large White sow populations in an evaluation of litter size and stayability traits

Trait1)	Landrace					Large White				
	
	N	Mean	SD	Min	Max	N	Mean	SD	Min	Max
AFF (d)	10,794	367	26.0	311	442	9,475	377	27.3	304	438
NBA1	10,794	9.38	2.65	1	18	9,475	9.95	2.84	1	20
NBA2	9,601	9.97	2.53	1	20	8,390	10.67	2.72	1	20
NBA3	8,836	10.54	2.50	1	20	7,772	11.13	2.67	1	21
STAY14	8,117	0.76	0.43	0	1	7,081	0.75	0.43	0	1

SD, standard deviation.

1) AFF, age at first farrowing; NBA1, number of piglets born alive in the first parity; NBA2, number of piglets born alive in the second parity; NBA3, number of piglets born alive in the third parity; NBA4, number of piglets born alive in the fourth parity; STAY14, stayability from first to fourth litter.

**Table 3:** Posterior distribution estimates for (co)variance components and genetic parameters (standard errors in brackets) for litter size (continuous traits) and stayability (threshold trait) from Thai Landrace and Large White sow populations using a linear-threshold model

Breed	Traits1)	Variance components2)			h^2^

		$\sigma_{a}^{2}$	$\sigma_{e}^{2}$	$\sigma_{p}^{2}$	
Landrace	NBA1	0.04 (0.00)	6.84 (0.00)	6.88 (0.00)	0.01 (0.00)
	NBA2	0.25 (0.01)	6.11 (0.01)	6.36 (0.00)	0.04 (0.01)
	NBA3	0.27 (0.00)	5.91 (0.00)	6.18 (0.00)	0.04 (0.01)
	STAY14	0.05 (0.00)	1.00 (0.00)	1.05 (0.00)	0.05 (0.01)
Large White	NBA1	0.17 (0.04)	7.48 (0.12)	7.65 (0.11)	0.02 (0.01)
	NBA2	0.36 (0.09)	6.88 (0.13)	7.24 (0.12)	0.05 (0.01)
	NBA3	0.44 (0.09)	6.60 (0.13)	7.04 (0.12)	0.06 (0.01)
	STAY14	0.05 (0.01)	1.00 (0.02)	1.05 (0.03)	0.05 (0.01)

1) NBA1, number of piglets born alive in the first parity; NBA2, number of piglets born alive in the second parity; NBA3, number of piglets born alive in the third parity; STAY14, stayability from first to fourth litter.

2) $\sigma_{a}^{2}$, additive genetic variance; $\sigma_{e}^{2}$, residual variance; $\sigma_{p}^{2}$, phenotypic variance; h^2^, heritability.

**Table 4:** Posterior means for heritabilities (±SE; diagonal), genetic (±SE; above diagonal), and phenotypic (±SE; below diagonal) correlations between litter size (continuous traits) and stayability (threshold trait) from a four-trait analysis and across parities from Thai Landrace sows using a linear-threshold model

Traits1)	NBA1	NBA2	NBA3	STAY14
NBA1	**0.01 (0.00)**	0.73 (0.13)	0.89 (0.05)	0.65 (0.16)
NBA2	0.12 (0.01)	0.**04 (0.01)**	0.85 (0.08)	0.59 (0.18)
NBA3	0.11 (0.01)	0.15 (0.01)	**0.04 (0.01)**	0.43 (0.18)
STAY14	0.08 (0.01)	0.09 (0.01)	0.12 (0.02)	**0.05 (0.01)**

SE, standard error.

1) NBA1, number of piglets born alive in the first parity; NBA2, number of piglets born alive in the second parity; NBA3, number of piglets born alive in the third parity; STAY14, stayability from first to fourth litter.

**Table 5:** Posterior mean for heritabilities (±SE; diagonal), genetic (±SE; above diagonal) and phenotypic (±SE; below diagonal) correlations between litter size (continuous traits) and stayability (threshold trait) from a four-trait analysis and across parities from Thai Large White sows using a linear-threshold model

Traits1)	NBA1	NBA2	NBA3	STAY14
NBA1	**0.02 (0.01)**	0.72 (0.01)	0.92 (0.02)	0.55 (0.01)
NBA2	0.10 (0.01)	**0.05 (0.01)**	0.86 (0.03)	0.12 (0.01)
NBA3	0.13 (0.01)	0.18 (0.01)	**0.06 (0.01)**	0.33 (0.01)
STAY14	0.06 (0.01)	0.05(0.01)	0.13 (0.02)	**0.05 (0.01)**

SE, standard error.

1) NBA1, number of piglets born alive in the first parity; NBA2, number of piglets born alive in the second parity; NBA3, number of piglets born alive in the third parity; STAY14, stayability from first to fourth litter.
